# A systematic review and meta-analysis of the effect of hyperglycemia on admission for acute myocardial infarction in diabetic and non-diabetic patients

**DOI:** 10.1186/s13098-024-01459-w

**Published:** 2024-09-12

**Authors:** Reem Alawaji, Mohammed Musslem, Emtenan Alshalahi, Abdulaziz Alanzan, Albaraa Sufyani, Maram Alhati, Alhanouf Almutairi, Mahdi Alqaffas, Batool Alattas, Adhari Alselmi

**Affiliations:** 1 Clinical Sciences Department, MBBS program, Fakeeh College for Medical Sciences, Jeddah, Saudi Arabia; 2 University of Jeddah, Jeddah, Saudi Arabia; 3 Qassim University, Buraydah, Saudi Arabia; 4 Majmaah University, Al Majma’ah, Saudi Arabia; 5 King Abdualziz University, Jeddah, Saudi Arabia; 6 Sulaiman Alrajhi University, Al Bukayriyah, Saudi Arabia; 7 Medical University of Lodz, Lodz, Poland; 8 Imam Abdulrahman Bin Faisal University, Dammam, Saudi Arabia; 9 Dr.Sulaiman Fakeeh Medical Center, Jeddah, Saudi Arabia

**Keywords:** Diabetes, Acute myocardial infarction, Glucose, Hyperglycemia

## Abstract

**Introduction:**

Regarding a potential relationship between diabetes and the prognostic significance of hyperglycemia in patients presenting with acute myocardial infarction (AMI), there is still debate. Therefore, we aimed in this study to demonstrate the effect of hyperglycemia on different outcomes in AMI patients, whether they are diabetic or not.

**Methods:**

We searched PubMed, Web of Science, and Scopus using the following search strategy: “Diabetes” or “Diabetic” AND “Acute myocardial infarction” OR “AMI” AND “hyperglycemia” OR “glucose level” to find eligible articles that needed to go through the screening process for inclusion in our study. We conducted a meta-analysis of 19 included studies from Japan, Germany, China, the United Kingdom, and others using Review Manager version 5.4 software, pooling the mean difference in continuous variables, the number and total of dichotomous variables to measure the odds ratio (OR), and the generic inverse variance of OR or hazard ratio (HR) as reported in the included studies.

**Results:**

The mean age of the participants ranged from 56.3 to 72.3 years old. The difference in blood glucose levels between diabetes and non-diabetes patients was found to be statistically significant, with an SMD of 1.39 (95%CI: 1.12, 1.66, *p* < 0.00001). In diabetic patients, hyperglycemia was statistically significantly associated with mortality, with a HR of 1.92 (95% CI: 1.45, 2.55, *p* < 0.00001) and an OR of 1.76 (95% CI: 1.15, 2.7, *p* = 0.01). In non-diabetic patients admitted with AMI, hyperglycemia was statistically significantly associated with mortality, with a HR of 1.56 (95% CI: 1.31, 1.86, *p* < 0.00001) and an OR of 2.89 (95% CI: 2.47, 3.39, *p* < 0.00001). AMI patients who were diabetic were statistically more likely to have a major adverse cardiovascular event (MACE) (HR = 1.9; 95% CI: 1.19–3.03; *p* = 0.007). AMI patients who were not diabetic were also statistically more likely to have a MACE (HR = 1.6; 95% CI: 1.15–2.23, *p* = 0.006).

**Conclusion:**

Hyperglycemia in AMI patients is a predictor of worse outcomes, including MACE and mortality, regardless of whether these patients are diabetic or not. In these patients, some factors act as predictors of mortality, including older age, higher glucose levels on admission, and a high Killip class.

**Supplementary Information:**

The online version contains supplementary material available at 10.1186/s13098-024-01459-w.

## Introduction

Globally, acute coronary syndromes (ACS) constitute a major cause of mortality, with acute myocardial infarction (AMI) being particularly concerning due to its high short- and long-term death rates [1]. The World Health Organization (WHO) predicts that by 2030, there will be over 23.6 million cardiovascular deaths worldwide, marking a significant increase from previous decades [2]. Even in the absence of preexisting diabetes, hyperglycemia can emerge during an AMI due to stress-induced increases in catecholamines, steroids, and glucagon levels, along with a decrease in insulin levels [3].

According to previous studies, 20 to 50% of patients with ST-segment elevation myocardial infarction (STEMI) experience stress hyperglycemia upon admission [4, 5]. The American Heart Association and the Endocrine Society Clinical Guidelines define stress hyperglycemia as a random plasma glucose level above 140 mg/dL in both diabetic and non-diabetic hospitalized patients [6]. A study has highlighted that hyperglycemia, whether in diabetic or non-diabetic patients, adversely affects AMI outcomes [7].

Research has shown that type 2 diabetes mellitus (T2DM) is a common comorbidity among patients with cardiovascular diseases, particularly AMI, and is detected in more than 20% of patients admitted for suspected AMI [6]. T2DM is associated with double the risk of in-hospital mortality and increases the likelihood of major adverse cardiovascular events (MACE) during follow-up [6]. Additionally, 10–20% of non-diabetic AMI patients exhibit significant hyperglycemia, which is linked to a higher risk of MACE [7]. Admission hyperglycemia is recognized as an independent predictor of poor short- and long-term outcomes in AMI patients [8].

A study examining the prognostic significance of the stress hyperglycemia ratio (SHR) and admission blood glucose (ABG) levels in AMI patients found that elevated SHR and ABG levels are associated with increased 30-day and 1-year mortality, especially in diabetic patients [9]. For instance, Meshref [10] noted that hyperglycemia correlates with larger infarct sizes, greater summation of ST-segment elevation (sum STE), maximum ST-segment elevation (max STE), higher echocardiographic wall motion score index (WMSI), and a lower segmental ejection fraction (EF).

Hyperglycemia generally increases the incidence of MACE, including re-hospitalization for heart failure, stroke, and coronary disease, in addition to raising mortality rates [11]. Regardless of whether thrombolysis or primary percutaneous coronary intervention (pPCI) is used as reperfusion therapy, hyperglycemia at admission is a significant predictor of adverse outcomes in AMI patients [12, 13]. Therefore, this study aims to demonstrate the effect of hyperglycemia on different outcomes in AMI patients, whether they are diabetic or not.

## Methods

Adhering to the Cochrane Handbook of Systematic Reviews of Interventions at each step [14] and following the Preferred Reporting Items for Systematic Reviews and Meta-Analyses (PRISMA) statement’s guidelines, we conducted this systematic review and meta-analysis [15].

### Database searching and screening

Using the following search strategy: “Diabetes” or “Diabetic” AND “Acute myocardial infarction” OR “AMI” AND “hyperglycemia” OR “glucose level,” we searched PubMed, Web of Science, and Scopus for eligible articles that should undergo the screening process to determine their ability to be included in our study. After searching the database, we removed the duplicates from the resulting articles using EndNote version 7 [16]. software, and then we uploaded the remaining articles on Rayyan software [17] to conduct the screening process. First, four authors who worked independently conducted the screening by title and abstract to see the eligibility for inclusion. Then they conducted full-text screening of the included articles from the previous step. Any conflicts were referred to a senior author to resolve.

### Inclusion and exclusion criteria

The predetermined inclusion and exclusion criteria used for screening were any observational (cohort, cross-sectional, or case-control) and randomized controlled trials (RCT) investigating the effects of hyperglycemia in diabetic or non-diabetic AMI patients on short- or long-term outcomes such as mortality and the occurrence of MACE. We excluded studies that didn’t measure the effect of hyperglycemia, populations other than AMI, case reports, case series, and reviews.

### Quality assessment

For the included observational cohort studies, we used the New Castle Ottawa scale tool provided by Cochrane for the assessment of quality. It is composed of eight questions with a maximum of one star for each, except for the comparability question, which can get two stars. Therefore, the highest score is nine, while the lowest is zero. Studies scoring from 0 to 3 were considered of low quality, 4–6 were of moderate quality, and 7–9 were of high quality [18].

### Data extraction

Using Microsoft Excel sheets, four independent authors conducted the process of data extraction to extract the baseline data (study design, country, sample size, groups, age, and gender) in addition to the outcomes (blood glucose on admission, odds ratio [OR], hazard ratio [HR] of mortality and MACE, mortality rate, factors affecting mortality including age, admission glucose levels, and Killip class) of the included studies. Any differences or conflicts were resolved by a senior author.

### Statistical analysis and sensitivity analysis

Using Review Manager version 5.4 software, we conducted the meta-analysis of the included studies by pooling the mean difference in continuous variables, the number and total of dichotomous variables to measure the OR, and the generic inverse variance of OR or HR as they were reported in the included studies. The results were considered statistically significant at a p-value of less than or equal to 0.05. The confidence interval (CI) was 95%, and the I2 was used to test the heterogeneity with the p-value for significance. Using OpenMetaAnalyst software, we conducted a sensitivity analysis using the leave-one-out method to remove the studies that caused heterogeneity in the heterogeneous outcomes.

## Results

### Database searching and screening

**Fig. 1:**
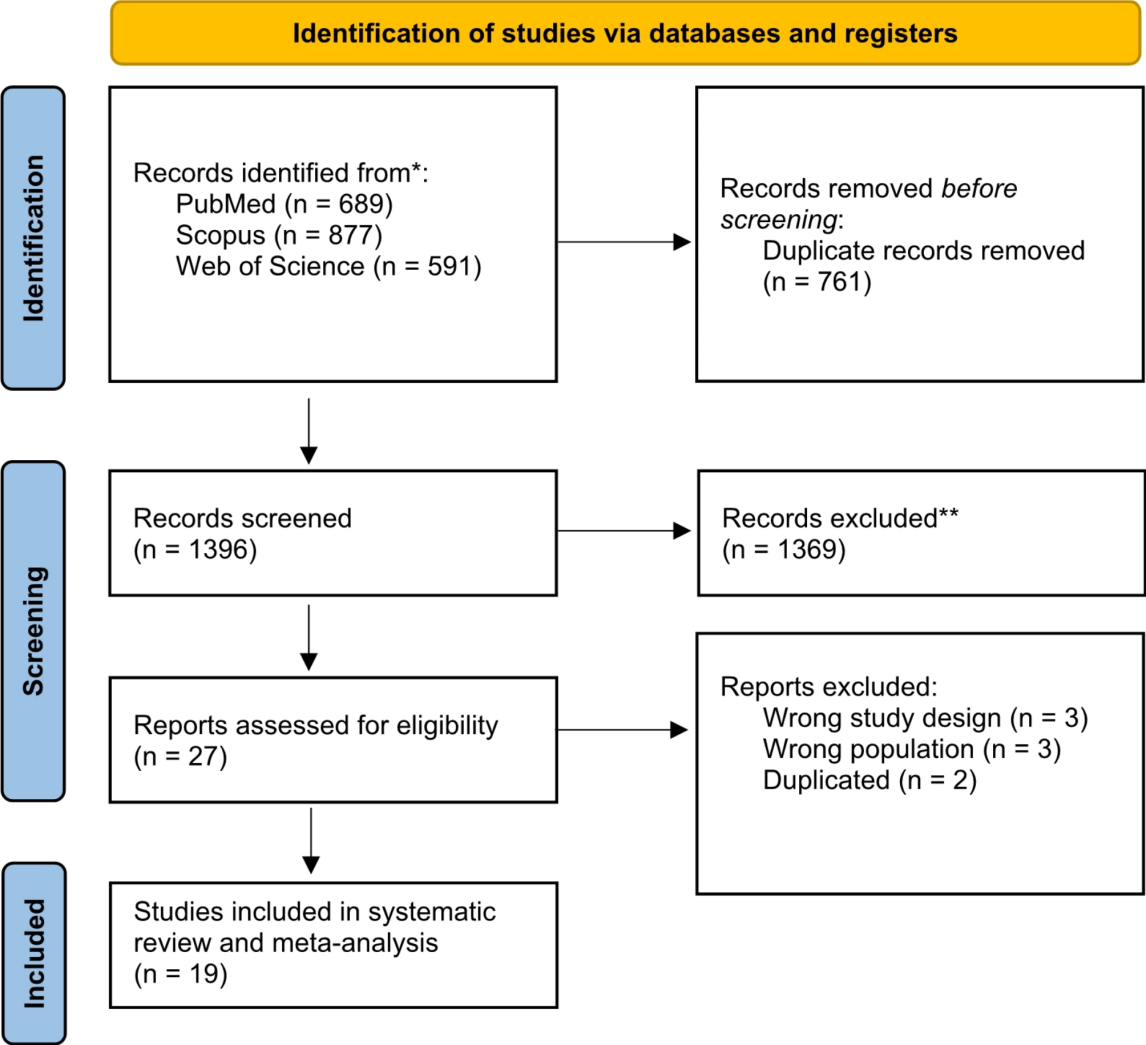
PRISMA flow diagram of database searching and screening

The database searching process yielded a total of 2157 articles with 761 duplicates, so a total of 1396 articles entered the title and abstract screening. A total of 1369 articles were excluded, and then 27 articles were screened in full text to yield a total of 19 articles (6,19–36) for the meta-analysis (Fig. 1).

### Quality assessment

In terms of ascertainment of exposure, all studies employed reliable methods to measure hyperglycemia, typically using hospital records or standardized glucose tests. For instance, two studies ensured accurate hyperglycemia measurement through validated laboratory tests. The studies demonstrated that the outcome of interest (e.g., mortality or MACE) was not present at the start of the study. This was evidenced by baseline assessments ensuring participants were free of these outcomes upon enrollment. Comparability of cohorts was achieved in most studies by controlling for key confounders such as age, gender, and baseline health conditions. Some studies employed multivariate analyses to adjust for these variables, enhancing the reliability of their findings. Regarding the assessment of outcomes, all studies used robust methods to track mortality and MACE, often through follow-up visits and hospital records. The follow-up durations were generally adequate, with most studies ensuring sufficient time to observe significant outcomes. The adequacy of follow-up was also well-maintained in most studies, with low drop-out rates and thorough accounting for missing data. This thorough follow-up contributed to the high-quality ratings for studies. The overall high quality of the included studies reinforces the reliability of our findings.

The risk of bias assessment results for all studies are shown in (Table 1); out of the 19 studies, 13 studies were considered of high quality (scores 7–9), while only six studies were considered of moderate quality (scores 4–6). High-quality studies provided robust data with minimal bias, ensuring reliability in our meta-analysis results. Moderate-quality studies, although slightly limited in some aspects, still contributed valuable data.

**Table 1:** Quality assessment of the included cohort studies using New Castle Ottawa Scale

Study name	Representativeness of the exposed cohort (★)	Selection of the non exposed cohort (★)	Ascertainment of exposure (★)	Demonstration that outcome of interest was not present at start of study (★)	Comparability of cohorts on the basis of the design or analysis (max★★)	Assessment of outcome (★)	Was follow-up long enough for outcomes to occur? (★)	Adequacy of follow up of cohorts (★)	Quality level
Upur 2022. [22]	★	0	★	★	★★	★	★	0	High (7)
Paolisso 2021. [6]	★	★	0	★	★★	★	★	0	High (7)
Ritsinger 2021. [36]	0	0	★	0	★★	★	★	★	Moderate (6)
Ding 2019. [35]	★	★	★	0	★★	★	★	★	High (8)
Schmitz 2022. [23]	0	0	★	★	★★	★	★	0	Moderate (6)
Cui 2022. [34]	★	★	★	★	★★	★	★	★	High (9)
Kojima 2019. [33]	★	★	★	0	★★	★	★	★	High (8)
Thoegersen 2020. [32]	★	★	★	★	★★	★	0	★	High (8)
Demarchi 2020. [31]	★	★	0	★	★	★	★	0	Moderate (6)
Ritsinger 2019. [30]	★	★	0	0	★★	0	★	★	Moderate (6)
Mamadjanov 2021. [29]	★	★	0	★	★	★	★	0	Moderate (6)
Cui 2021. [21]	★	★	0	★	★	★	★	0	Moderate (6)
Zhou 2020. [28]	★	★	0	0	★★	★	★	★	High (7)
Ferreira 2021. [27]	★	★	★	★	★★	0	0	★	High (7)
Jomaa 2018. [24]	★	★	★	★	★★	★	0	0	High (7)
Chattopadhyay 2018. [26]	★	★	★	★	★★	★	★	0	High (8)
Chattopadhyay 2019. [25]	★	★	★	★	★★	0	★	★	High (8)
Yuan 2022. [20]	★	★	★	★	★★	★	0	0	High (7)
Cui 2023. [19]	★	★	★	★	★★	0	★	0	High (7)

### Baseline characteristics

All 19 included articles were cohort studies conducted across various countries, including China, Italy, Sweden, Germany, Denmark, Japan, Portugal, Tunisia, and the United Kingdom, with sample sizes ranging from as few as 60 to as many as 10,094 participants, totaling 29,659 participants in this meta-analysis. Most studies compared hyperglycemic AMI patients with diabetes to those without diabetes, while others compared either of these groups against patients with no hyperglycemia and no diabetes. The mean age of participants ranged from 56.3 to 72.3 years, with a predominance of male participants in most studies, ranging from 48 to 82%. The largest cohort was observed in a Swedish study with 10,094 participants, whereas the smallest cohort, from China, involved 60 participants. Notably, one study also analyzed participants with Myocardial Infarction with Obstructive Coronary Arteries (MIOCA) and Myocardial Infarction with Non-Obstructive Coronary Arteries (MINOCA), providing a comprehensive overview of the impact of hyperglycemia across different myocardial infarction subtypes. The detailed baseline characteristics of the participants and the specific variables analyzed in each study, can be viewed in Table 2.

**Table 2:** Summary and baseline characteristics of the included studies. MIOCA = myocardial infraction with obstructive coronary arteries, MINOCA = myocardial infraction with non-obstructive coronary arteries

Study ID	Design	Country	Group 1	Group 2	Sample size		Age		Male, *n*(%)	
					Group 1	Group 2	Group 1	Group 2	Group 1	Group 2
Upur 2022. [22]	Cohort	China	Hyperglycemia and no diabetes	Hyperglycemia and diabetes	1179	425	56.3 (12.3)		965 (82)	279 (66)
Paolisso 2021. [6]	Cohort	Italy	MIOCA with hyperglycemia	MINOCA with hyperglycemia	877	38	72.3 (13.4)	74 (10.8)	615 (70)	12 (31.6)
Ritsinger 2021. [36]	Cohort	Sweden	Hyperglycemia and no diabetes	No hyperglycemia or diabetes	10,094	103	70 (23.7)	64.7 (27.1)	6927 (68.6	76 (73.8)
Ding 2019. [35]	Cohort	China	No hyperglycemia or diabetes	Hyperglycemia and no diabetes	1216	112	64.8 (15.6)	65.3 (12)	930 (76.5)	86 (76.8)
Schmitz 2022. [23]	Cohort	Germany	Hyperglycemia and diabetes	Hyperglycemia and no diabetes	681	1630	68 (11)	63.7 (12.7)	481 (70.6)	1203 (73.8)
Cui 2022. [34]	Cohort	China	Hyperglycemia and diabetes	Hyperglycemia and no diabetes	1681	1006	62.9 (12)	61.4 (13.1)	1174 (69.8)	767 (76.2)
Kojima 2019. [33]	Cohort	Japan	Hyperglycemia and no diabetes	Hyperglycemia and diabetes	970	600	67.6 (12.6)	67.4 (9.7)	703 (72.5)	433 (72.3)
Thoegersen 2020. [32]	Cohort	Denmark	Hyperglycemia and no diabetes	Hyperglycemia and diabetes	1307	273	66.9 (12.20)	69.19 (9.95)	982 (75.1)	198 (72.5)
Demarchi 2020. [31]	Cohort	Italy	Hyperglycemia and no diabetes	Hyperglycemia and diabetes	2958		65.6 (12.6)		2248 (76)	
Ritsinger 2019. [30]	Cohort	Sweden	Hyperglycemia and no diabetes	Hyperglycemia and diabetes	198	1124	67 (11)		NR	
Mamadjanov 2021. [29]	Cohort	Germany	Hyperglycemia and no diabetes	Hyperglycemia and diabetes	3434	2096	65–84		3514 (63.5)	
Cui 2021. [21]	Cohort	China	Hyperglycemia and no diabetes	Hyperglycemia and diabetes	425	239	67.7 (13.2)	70.3 (11.3)	300 (70.6)	127 (53.3)
Zhou 2020. [28]	Cohort	China	Hyperglycemia and no diabetes	Hyperglycemia and diabetes	60	95	57.1 (10.6)	57.1 (11.5)	48 (80)	77 (81.1)
Ferreira 2021. [27]	Cohort	Portugal	Hyperglycemia and no diabetes	Hyperglycemia and diabetes	426	325	69.9 (12.9)	68.5 (11.2)	291 (68.5)	220 (67.7)
Jomaa 2018. [24]	Cohort	Tunisia	Hyperglycemia and no diabetes	Hyperglycemia and diabetes	865	464	60.39 (12.8)		997 (77.3)	
Chattopadhyay 2018. [26]	Cohort	United Kingdom	Hyperglycemia and no diabetes	No hyperglycemia or diabetes	172	165	69 (20)	61 (13)	122 (70.9)	120 (72.7)
Chattopadhyay 2019. [25]	Cohort	United Kingdom	Hyperglycemia and no diabetes	No hyperglycemia or diabetes	200	474	68 (14.6)	64 (12.6)	136 (68.0)	346 (73.0)
Yuan 2022. [20]	Cohort	China	Hyperglycemia and no diabetes	No hyperglycemia or diabetes	147	1856	65.6 (18.3)	64.7 (16.3)	109 (74.1)	1466 (79)
Cui 2023. [19]	Cohort	China	Hyperglycemia and no diabetes	Hyperglycemia and diabetes	3227	2081	61.3 (12.9)	63.1 (11.5)	2541 (78.7)	1420 (68.2)

### Meta-analysis

**Fig. 2:**
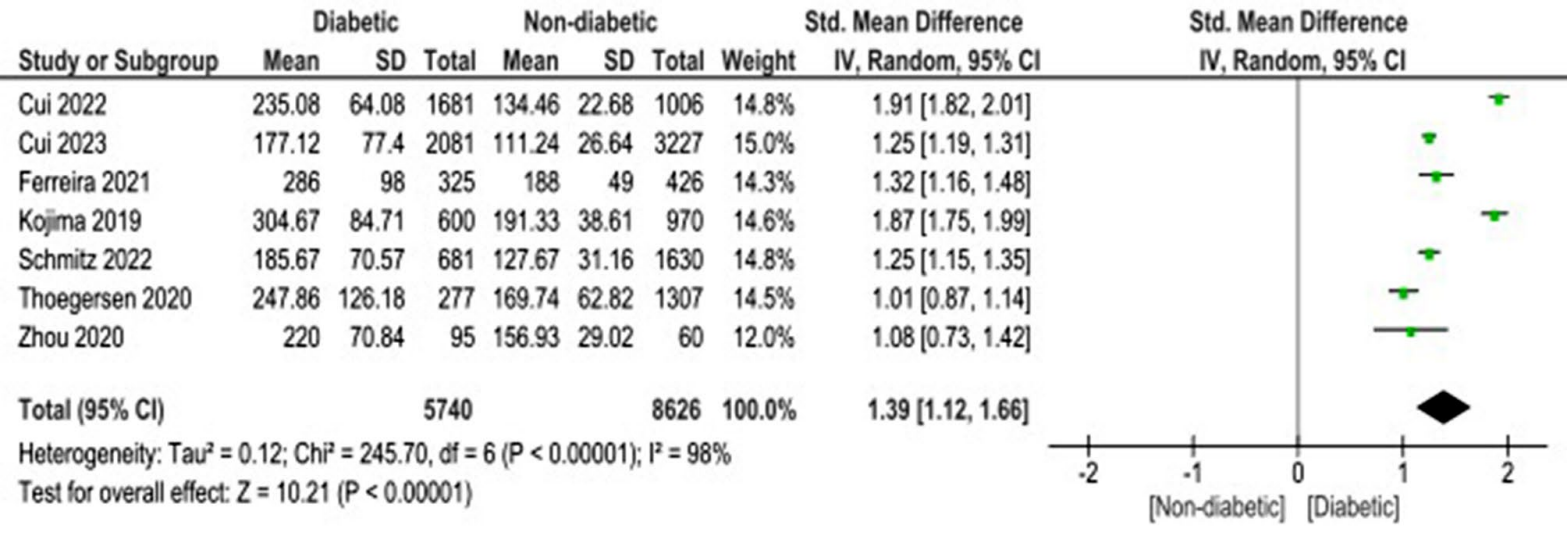
Comparison between admission blood glucose in diabetic vs. non-diabetic patients

The difference between diabetes and non-diabetes patients regarding blood glucose level was found to be statistically significant with SMD of 1.39 (95%CI: 1.12, 1.66, *p* < 0.00001) with heterogeneity (I2 = 98%, *p* < 0.00001) (Fig. 2).

**Fig. 3:**
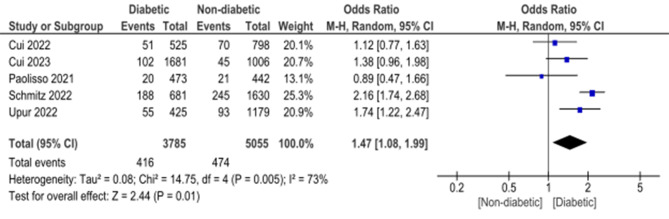
Comparison between mortality rate in diabetic and non-diabetic patients who have hyperglycemia on admission with acute myocardial infarction

Hyperglycemia in diabetes patients was found to be more statistically significantly associated with mortality compared to hyperglycemia in non-diabetic patients with OR of 1.47 (95%CI: 1.08, 1.99, *p* = 0.01) and heterogeneity measured by I2 = 73%, *p* = 0.005 (Fig. 3).

**Fig. 4:**
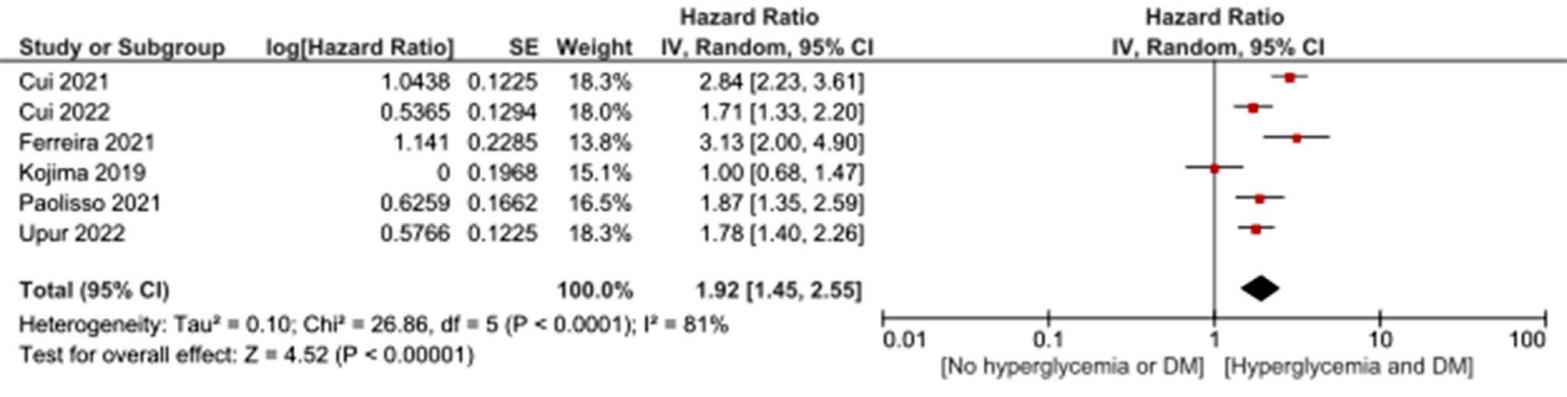
Association of hyperglycemia with mortality in diabetic patients admitted with acute myocardial infarction using hazard ratio

**Fig. 5:**
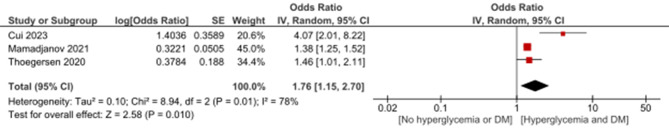
Association of hyperglycemia with mortality in diabetic patients admitted with acute myocardial infarction using odds ratio

Hyperglycemia in diabetic patients was statistically significantly associated with mortality with HR of 1.92 (95%CI: 1.45, 2.55, *p* < 0.00001) with heterogeneity (I2 = 81%, *p* < 0.0001). (Fig. 4) and OR of 1.76 (95%CI: 1.15, 2.7, *p* = 0.01) with heterogeneity (I2 = 78%, *p* = 0.01) (Fig. 5).

**Fig. 6:**
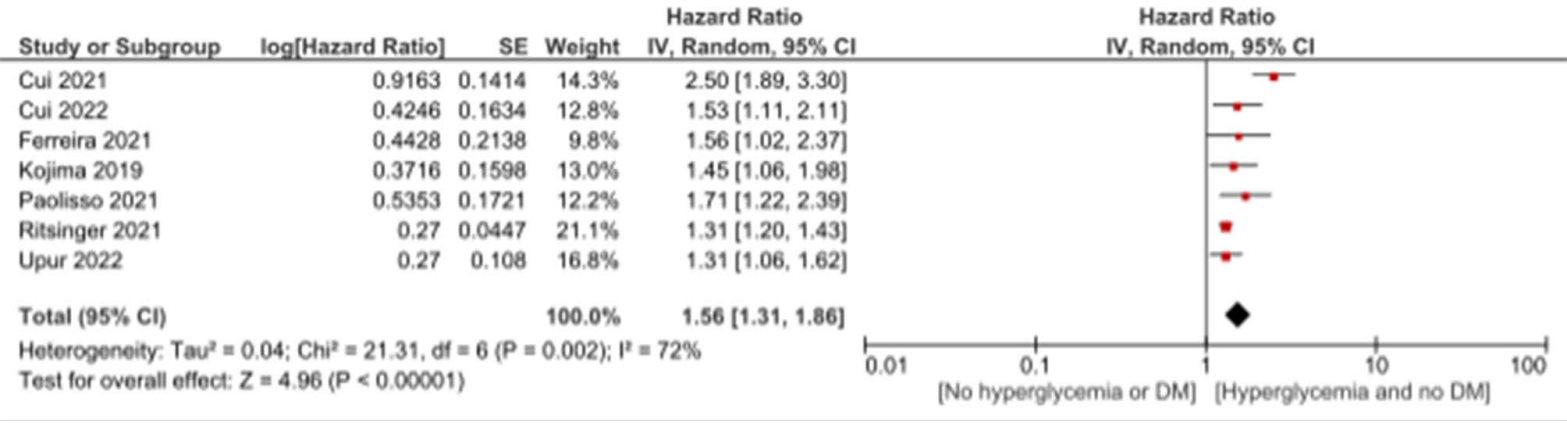
Association of hyperglycemia with mortality in non-diabetic patients admitted with acute myocardial infarction using hazard ratio

**Fig. 7:**
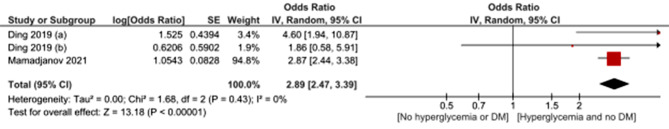
Association of hyperglycemia with mortality in non-diabetic patients admitted with acute myocardial infarction using odds ratio

In non-diabetic patients admitted with AMI, hyperglycemia was statistically significantly associated with mortality with HR of 1.56 (95%CI: 1.31, 1.86, *p* < 0.00001), heterogeneity (I2 = 72%, *p* = 0.002) and OR of 2.89 (95%CI: 2.47, 3.39, *p* < 0.00001) and no heterogeneity (I2 = 0%). (Figs. 6, and 7)

**Fig. 8:**
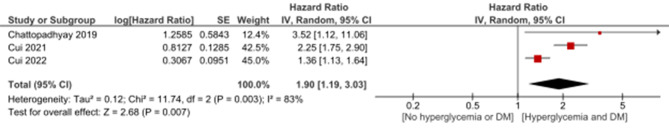
Association of hyperglycemia with MACE in diabetic patients admitted with acute myocardial infarction using hazard ratio

**Fig. 9:**
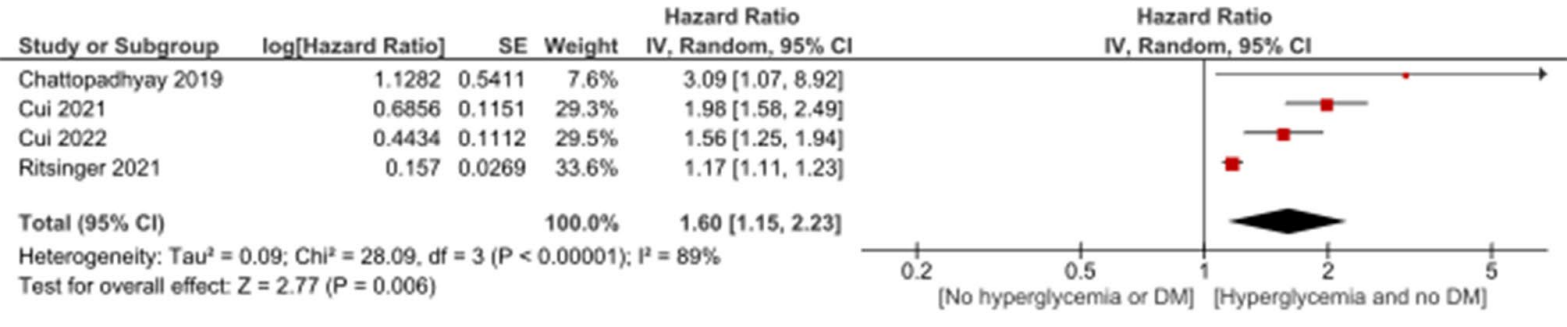
Association of hyperglycemia with MACE in non-diabetic patients admitted with acute myocardial infarction using hazard ratio

Moreover, hyperglycemia in diabetic patients admitted with AMI was statistically significantly associated with the occurrence of MACE with HR of 1.9 (95%CI: 1.19, 3.03, *p* = 0.007) and heterogeneity (I2 = 83%, *p* = 0.003) (Fig. 8). In addition, hyperglycemia in non-diabetic AMI patients was statistically significantly associated with the occurrence of MACE with HR of 1.6 (95%CI: 1.15, 2.23, *p* = 0.006) and heterogeneity (I2 = 89%, *p* < 0.00001) (Fig. 9).

**Fig. 10:**
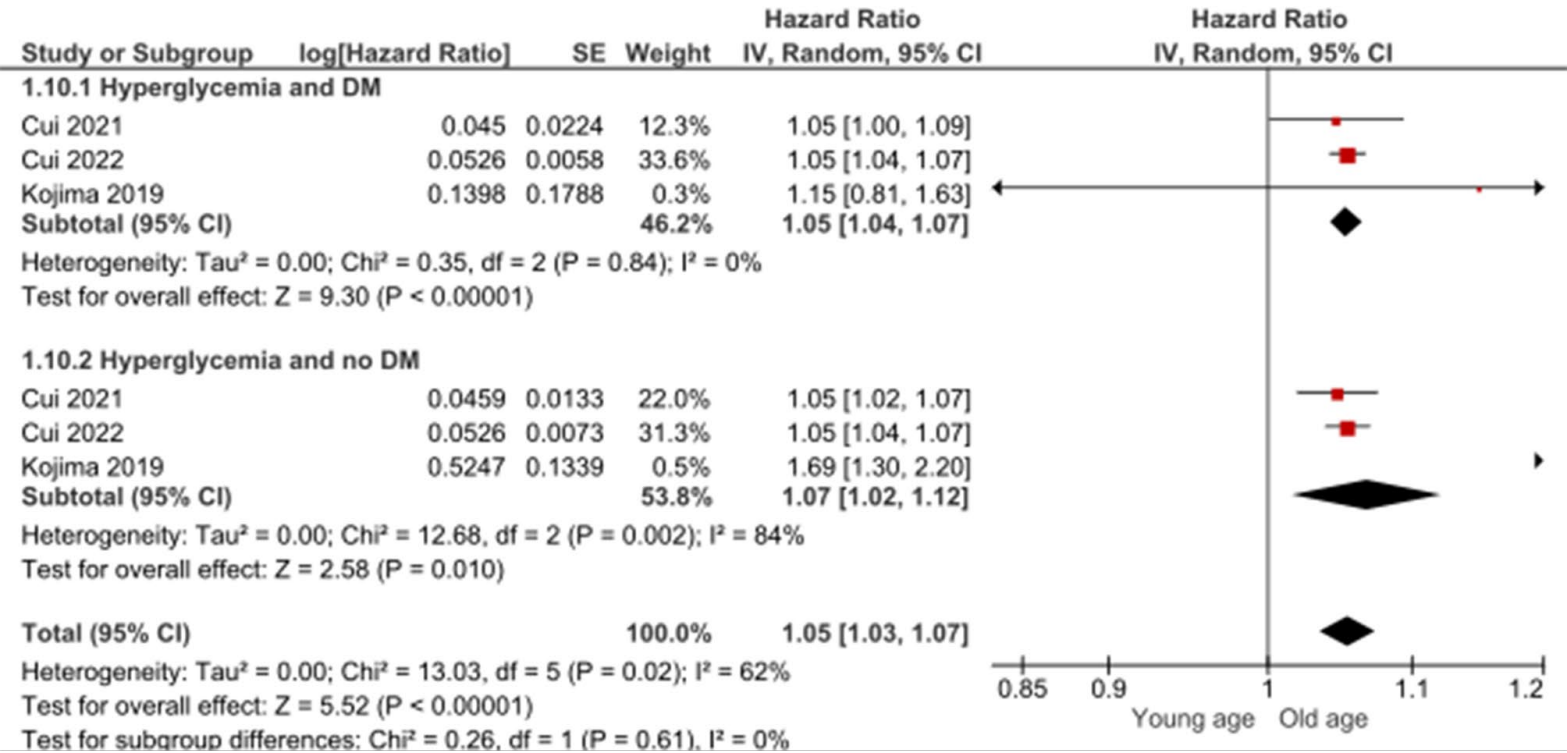
The effect of age on mortality in diabetic and non-diabetic acute myocardial infraction patients who have hyperglycemia

**Fig. 11:**
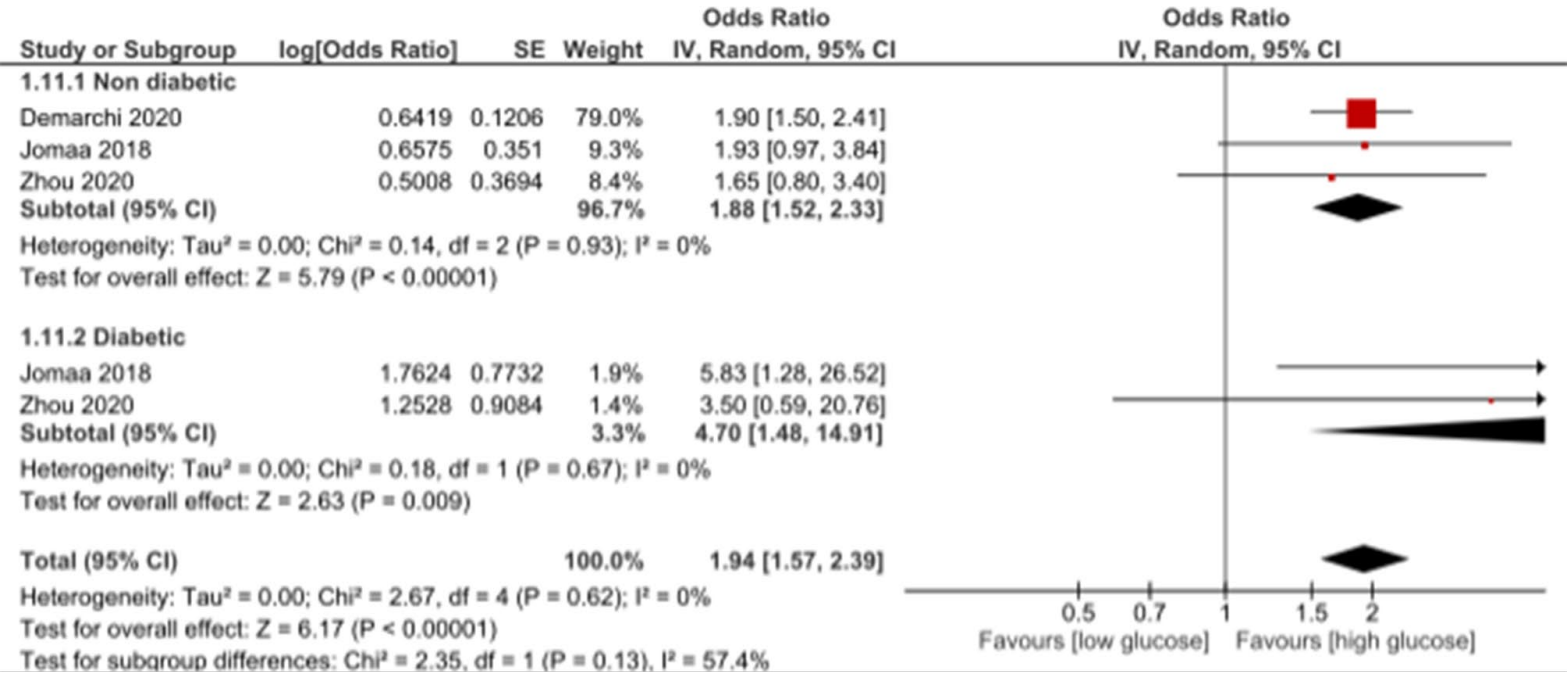
Association of admission glucose levels with mortality in diabetic and non-diabetic acute myocardial infarction patients

**Fig. 12:**
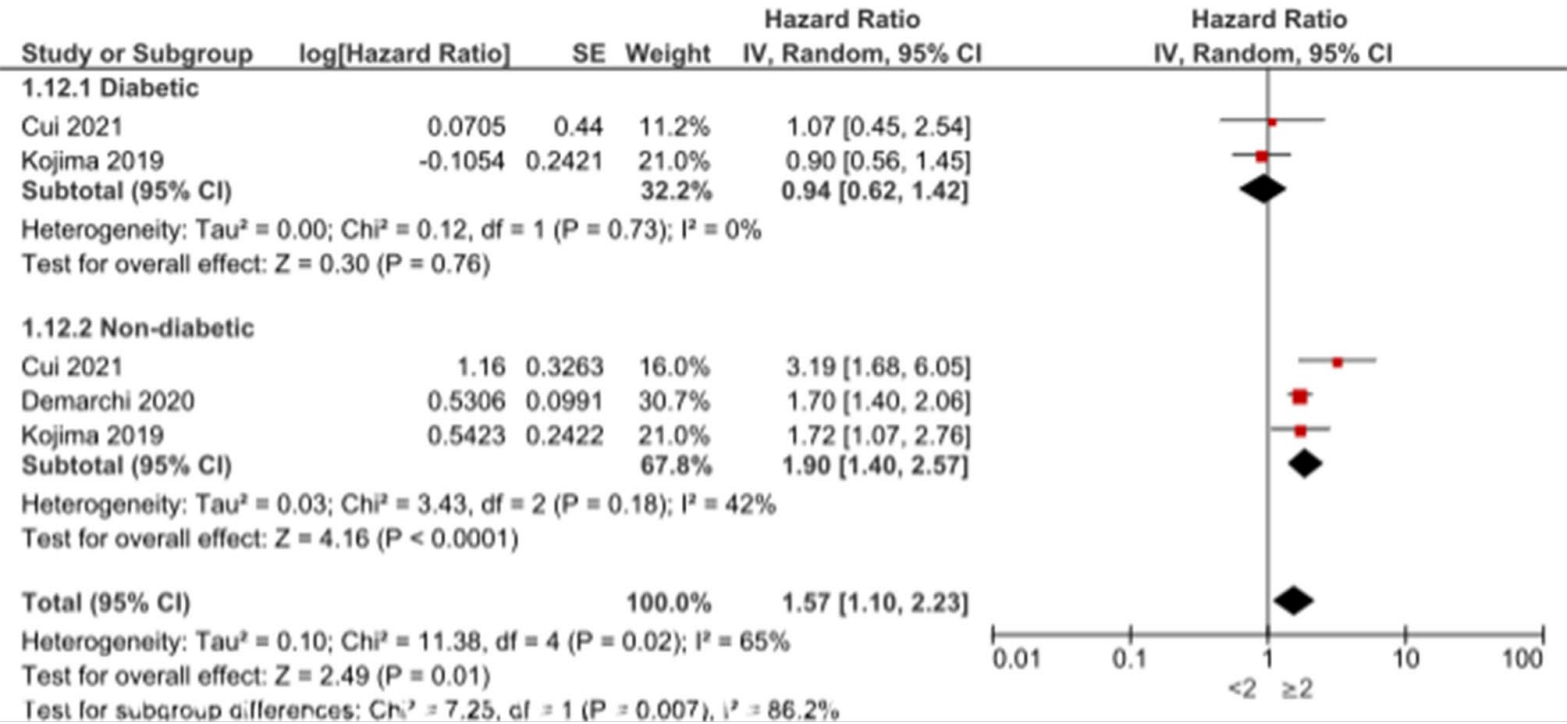
Association of Killip class with mortality in diabetic and non-diabetic acute myocardial infarction patients

Age was among the factors that predicted mortality after hyperglycemia in diabetic and non-diabetic AMI patients, with HR of 1.05 (1.04, 1.07, *p* < 0.00001) and no heterogeneity (I2 = 0%) in diabetic patients and HR of 1.07 (95%CI: 1.02, 1.12, *p* = 0.01) and heterogeneity (I2 = 84%, *p* = 0.01) in non-diabetic patients (Fig. 10). It was observed that increased glucose levels on admission are statistically significant predictors of mortality in diabetic and non-diabetic patients with OR of 4.7 (95%CI: 1.48, 14.91, *p* = 0.009) and no heterogeneity (I2 = 0%) and OR of 1.88 (95%CI: 1.52, 2.33, *p* < 0.00001) and no heterogeneity (I2 = 0%), respectively (Fig. 11). Killip class ≥ 2 was statistically significantly associated with mortality in non-diabetic AMI patients admitted with hyperglycemia with HR of 1.9 (95%CI: 1.4, 2.57, *p* < 0.0001) and non-significant heterogeneity (I2 = 42%, *p* = 0.18), while no significant association was observed between Killip class and mortality in diabetic patients with HR of 0.94 (95%CI: 0.62, 1.42, *p* = 0.76) (Fig. 12).

### Sensitivity analysis

After conducting a leave-one-out analysis for blood glucose levels comparison among the diabetic and non-diabetic patients, it was found that Cui et al. (2022) [34] and Kojima et al. (2019) [33] were the main sources of heterogeneity. (Supplementary Fig. 1)

For the comparison between the diabetic and non-diabetic patients regarding mortality, it was observed that Schmitz et al. (2022) [23] was the main source of heterogeneity. (Supplementary Fig. 2)

For the mortality outcome in diabetic patients using HR, Cui et al. (2021) [21] and Kojima et al. (2019) [33] were considered the main reasons for heterogeneity. (Supplementary Fig. 3) While using OR, Cui et al. (2023) [19] was the main source of heterogeneity. (Supplementary Fig. 4)

For the mortality outcome in non-diabetic patients using HR, Cui et al. (2021) [21] was considered the main source for heterogeneity. (Supplementary Fig. 5) Regarding the occurrence of MACE in diabetic patients, Cui et al. (2022) [34] caused heterogeneity in the outcome (Supplementary Fig. 6), while Ristinger et al. (2021) [36] caused heterogeneity in the MACE outcome of non-diabetics. (Supplementary Fig. 7) Yuan et al. (2022) [20] was observed to be the main cause of heterogeneity in the association of age with mortality using OR. (Supplementary Fig. 8).

## Discussion

The meta-analysis suggests a statistically significant difference in blood glucose levels between diabetes and non-diabetes patients. The observed standardized mean difference (SMD) of 1.39 in blood glucose levels between diabetes and non-diabetes patients is consistent with findings from previous studies [37, 38]. This reinforces the notion that diabetes patients tend to exhibit significantly higher blood glucose levels compared to their non-diabetic counterparts. However, it is crucial to note that our meta-analysis revealed high heterogeneity, indicating substantial variability across the included studies. This starkly contrasts with the comparatively lower heterogeneity reported in the studies by Redondo et al. (2020) [39]. While our findings align with prior research, the substantial heterogeneity underscores the need for a nuanced interpretation and calls for further investigation into factors contributing to the observed differences among studies. Addressing these variations may enhance the reliability and generalizability of conclusions drawn from future meta-analyses in this domain.

The analysis indicates a higher risk of mortality among diabetes patients with hyperglycemia compared to non-diabetic individuals. The odds ratio (OR) for mortality in diabetes patients with hyperglycemia is 1.47. Despite moderate heterogeneity, the statistical significance of the odds ratio suggests a noteworthy association. This finding implies that hyperglycemia in diabetes patients may be linked to an increased risk of mortality [40, 41]. The presence of moderate heterogeneity emphasizes the need for caution in interpretation, prompting further exploration into potential sources of variability among the included studies.

The hazard ratio (HR) of 1.92, indicating a substantial increase in mortality risk associated with hyperglycemia in diabetic patients, aligns with findings from prior research [42, 43]. However, the observed substantial heterogeneity suggests a notable variability among the included studies, emphasizing the importance of carefully considering potential sources of this heterogeneity for a more nuanced interpretation. Similarly, the odds ratio (OR) of 1.76 underscores an increased risk of mortality in diabetic patients with hyperglycemia, consistent with the results of studies [44, 45]. Nevertheless, the considerable heterogeneity warrants caution in interpreting these results, urging researchers to explore the underlying causes of this variability for more robust conclusions.

In non-diabetic patients admitted with acute myocardial infarction (AMI), hyperglycemia is associated with a statistically significant increase in mortality, as evidenced by a hazard ratio (HR) of 1.56. The presence of heterogeneity emphasizes the importance of carefully considering potential contributing factors to this variability. This association aligns with research by Sachdeva et al. (2020), which reported similar trends in non-diabetic AMI patients [46]. Furthermore, the odds ratio (OR) for mortality in this context is notably higher at 2.89, and interestingly, no heterogeneity is observed. The absence of heterogeneity in the odds ratio contrasts with the variability seen in the hazard ratio, indicating a more consistent pattern of increased mortality risk associated with hyperglycemia in this specific context.

In diabetic patients admitted with acute myocardial infarction (AMI), hyperglycemia is consistently associated with a higher risk of major adverse cardiac events (MACE), in line with findings from previous studies [47, 48]. However, the substantial heterogeneity observed in this subgroup suggests caution in interpreting results and prompts further investigation into potential contributing factors. Similarly, in non-diabetic AMI patients, hyperglycemia is significantly linked to an increased occurrence of MACE, aligning with research by Jensen et al. (2021) [49]. The observed high heterogeneity in this subgroup underscores the need for careful consideration of variability among studies. The presence of heterogeneity emphasizes the importance of understanding and addressing variations in study characteristics for more accurate clinical implications and interventions. Future research should focus on revealing the sources of heterogeneity to enhance the precision and applicability of these findings in diverse patient populations.

In both diabetic and non-diabetic acute myocardial infarction (AMI) patients, age stands out as a significant predictor of mortality following hyperglycemia. The small but significant hazard ratio (HR) of 1.05 in diabetic patients indicates a slight increase in mortality risk per unit increase in age, with no observed heterogeneity, providing confidence in this association. Conversely, in non-diabetic AMI patients, the HR of 1.07 suggests a slightly stronger impact of age on mortality. However, the substantial heterogeneity underscores the importance of considering potential variations in study characteristics. The association between age and mortality in diabetic AMI patients aligns with studies by Tachkov et al. (2020) [50]. In non-diabetic AMI patients, similar trends have been reported by Jansson et al. (2010) [51]. Notably, the presence of heterogeneity in the non-diabetic subgroup emphasizes the need for cautious interpretation and further exploration into potential sources of variability. While the association is more straightforward in diabetic patients, addressing heterogeneity in non-diabetic patients is crucial for refining our understanding of the age-related mortality risk and informing tailored clinical approaches.

Elevated glucose levels on admission were identified as significant predictors of mortality in both diabetic and non-diabetic patients. In diabetic patients, the odds ratio (OR) of 4.7 and in non-diabetic patients, the OR of 1.88 highlight the substantial impact of hyperglycemia on mortality risk. These findings align with studies by Kattel et al. (2017), emphasizing the consistent association between elevated glucose levels and increased mortality in diverse patient populations [52].

In non-diabetic AMI patients with hyperglycemia, Killip class ≥ 2 is significantly associated with mortality, in agreement with research by Mamadjanov et al. (2021) [29]. Conversely, no significant association between Killip class and mortality is observed in diabetic patients (HR: 0.94, 95% CI: 0.62, 1.42, *p* = 0.76), consistent with the findings of Hashmi et al. (2020) [53]. These interpretations underscore the universal impact of hyperglycemia on mortality risk in acute myocardial infarction patients while also emphasizing the divergent relevance of the Killip class in predicting outcomes based on diabetic status [54]. The insights from these comparisons contribute to a more nuanced understanding of risk factors and aid in tailoring interventions for diverse patient cohorts.

The sensitivity analysis highlighted significant sources of heterogeneity in various aspects of the study. In the comparison of blood glucose levels between diabetic and non-diabetic patients, Cui et al. (2022) [34] and Kojima et al. (2019) [33] were identified as primary contributors to heterogeneity. While the sources of heterogeneity are acknowledged, In the analysis of mortality between diabetic and non-diabetic patients, Schmitz et al. (2022) [23] emerged as the primary source of heterogeneity. Understanding the specific characteristics or methodologies contributing to this heterogeneity may facilitate a more nuanced interpretation of mortality outcomes in diabetic and non-diabetic groups. Examining mortality outcomes in diabetic patients using hazard ratios (HR), Cui et al. (2021) [21] and Kojima et al. (2019) [33] were identified as significant contributors to heterogeneity.

In the analysis using odds ratios (OR) for mortality in diabetic patients, Cui et al. (2023) [19] identified the main source of heterogeneity. Understanding the distinct factors introduced by this study can aid in refining interpretations and addressing potential biases. For mortality outcomes in non-diabetic patients using HR, Cui et al. (2021) [21] were pinpointed as the primary source of heterogeneity. Exploring how this study differs from others in the analysis may offer insights into the observed variations in non-diabetic mortality outcomes. Regarding major adverse cardiac events (MACE) in diabetic patients, Cui et al. (2022) [34] were recognized as the main contributors to heterogeneity. Understanding the specific aspects introduced by this study may help contextualize variations in MACE outcomes among diabetic patients.

In non-diabetic patients, Ristinger et al. (2021) [36] identified heterogeneity as the main source of heterogeneity in MACE outcomes. Previous studies may reveal distinct characteristics contributing to variations in MACE occurrences among non-diabetic individuals [55, 56]. Yuan et al. (2022) [20] observed age as the primary cause of heterogeneity in the association of age with mortality using odds ratios (OR). Understanding the unique aspects introduced by this study can contribute to a more comprehensive interpretation of age-related mortality associations.

A significant strength of this study is the inclusion of a large and diverse population across multiple countries, which enhances the generalizability of the findings. Additionally, the inclusion of studies analyzing both diabetic and non-diabetic populations, as well as different subtypes of myocardial infarction, provides a comprehensive understanding of the impact of hyperglycemia in various contexts. The study demonstrates that hyperglycemia is a strong predictor of negative outcomes, such as mortality and major adverse cardiovascular events (MACE). This comprehensive analysis substantiates the necessity of targeted interventions to manage hyperglycemia in AMI patients, further reinforcing the reliability of the findings.

However, this study has some limitations, including the fact that all included studies are observational in design, which carries a higher risk of bias compared to randomized controlled trials (RCTs). The reliance on retrospective data from diverse studies might introduce biases, affecting the overall robustness of the meta-analysis. Furthermore, the observed associations between hyperglycemia and mortality or major adverse cardiovascular events may be influenced by confounding factors not fully accounted for in the included studies. Therefore, while the findings are significant, they should be interpreted with caution. Further research, particularly randomized controlled trials (RCTs), is needed to refine these conclusions and determine effective interventions. Despite these limitations, our study contributes valuable evidence to understanding hyperglycemia’s role in AMI outcomes.

## Conclusion

Hyperglycemia in AMI patients are a predictor of worse outcomes including MACE, and mortality whether these patients are diabetic or not. Some factors act as predictors for mortality in these patients including older age, higher glucose levels on admission, and high Killip class. However, further studies are required to put a definite value for hyperglycemia and cut off for prognosis.

## Electronic supplementary material

Below is the link to the electronic supplementary material.

Supplementary Material 1

Supplementary Material 2

Supplementary Material 3

Supplementary Material 4

Supplementary Material 5

Supplementary Material 6

Supplementary Material 7

Supplementary Material 8

Supplementary Material 9

## Data Availability

All data generated or analyzed in this study are included in this publish article. For any further information or clarifications, please contact Alawaji, Reem (rzalawaji@gmail.com).
