# Correction: A systematic review and meta-analysis of the effect of hyperglycemia on admission for acute myocardial infarction in diabetic and non-diabetic patients

**DOI:** 10.1186/s13098-026-02224-x

**Published:** 2026-06-29

**Authors:** Reem Alawaji, Mohammed Musslem, Emtenan Alshalahi, Abdulaziz Alanzan, Albaraa Sufyani, Maram Alhati, Alhanouf Almutairi, Mahdi Alqaffas, Batool Alattas, Adhari Alselmi

**Affiliations:** 1 Clinical Sciences Department, MBBS program, Fakeeh College for Medical Sciences, Jeddah, Saudi Arabia; 2 University of Jeddah, Jeddah, Saudi Arabia; 3 Qassim University, Buraydah, Saudi Arabia; 4 Majmaah University, Al Majma’ah, Saudi Arabia; 5 King Abdualziz University, Jeddah, Saudi Arabia; 6 Sulaiman Alrajhi University, Al Bukayriyah, Saudi Arabia; 7 Medical University of Lodz, Lodz, Poland; 8 Imam Abdulrahman Bin Faisal University, Dammam, Saudi Arabia; 9 Dr.Sulaiman Fakeeh Medical Center, Jeddah, Saudi Arabia

**Correction: Diabetology & Metabolic Syndrome (2024) 16:224**

10.1186/s13098-024-01459-w

In this article [1], the author’s name Albaraa Sufyani was incorrectly written as Albarra Sufyani.

The original article has been corrected.
