# Supplementary material for: A systematic review and meta-analysis of the effect of hyperglycemia on admission for acute myocardial infarction in diabetic and non-diabetic patients
Source: Diabetol Metab Syndr. 2024 Sep 12;16:224. doi: 10.1186/s13098-024-01459-w (PMC11391676; doi:10.1186/s13098-024-01459-w)
Supplement: Supplementary file 1 — Supplementary Material 1 [file 13098_2024_1459_MOESM1_ESM.docx]

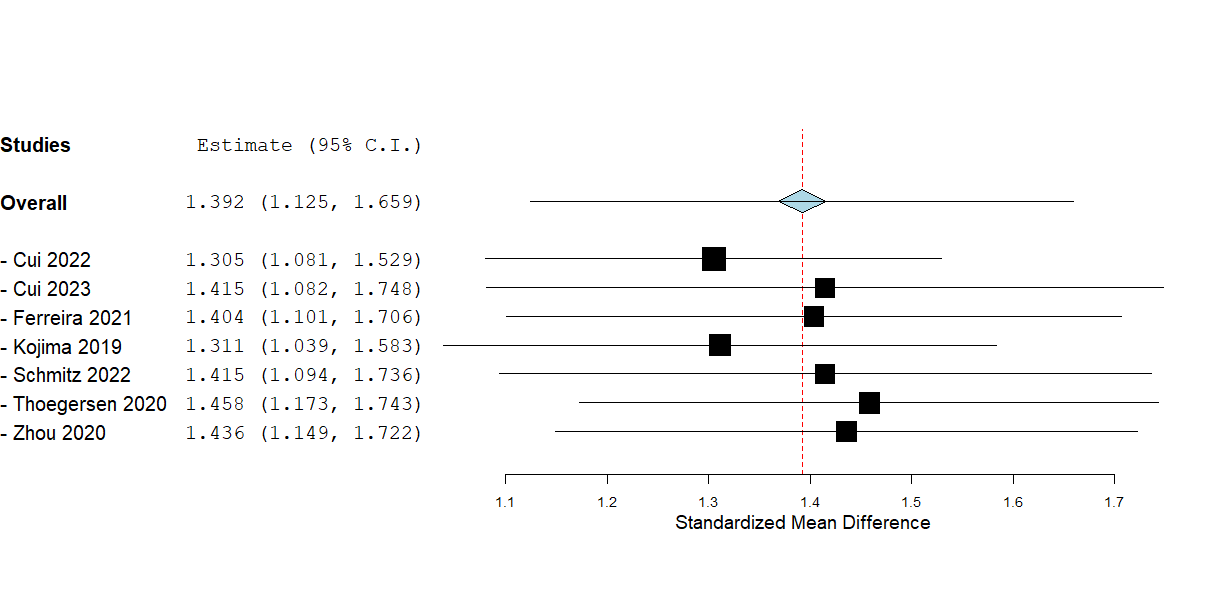


Supplementary figure 1: Leave-one-out analysis of the comparison between diabetic and non-diabetic patients regarding blood glucose


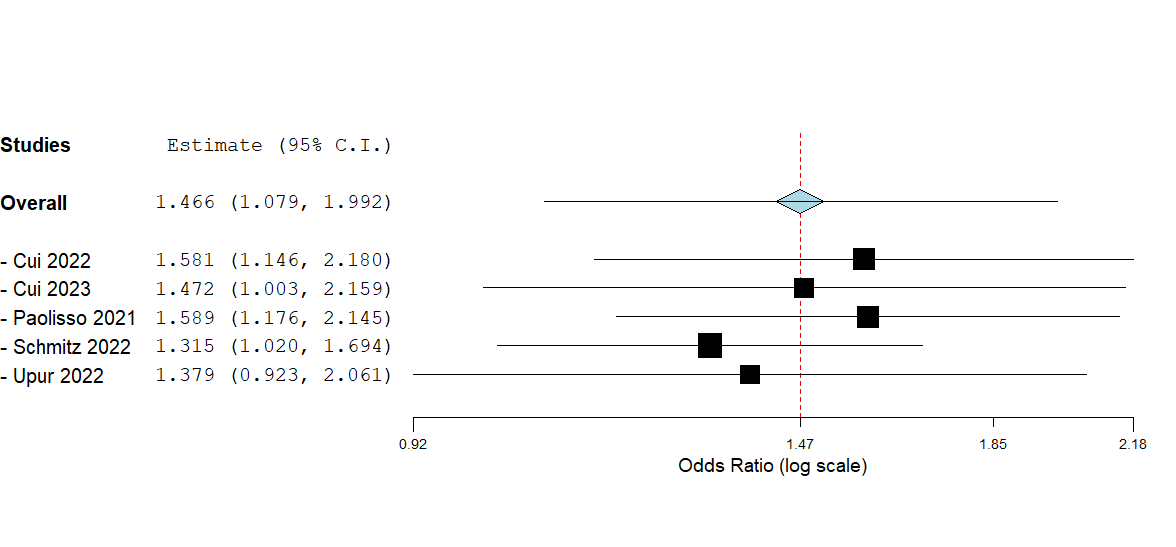


Supplementary figure 2: Leave-one-out analysis of the comparison between diabetic and non-diabetic patients regarding mortality


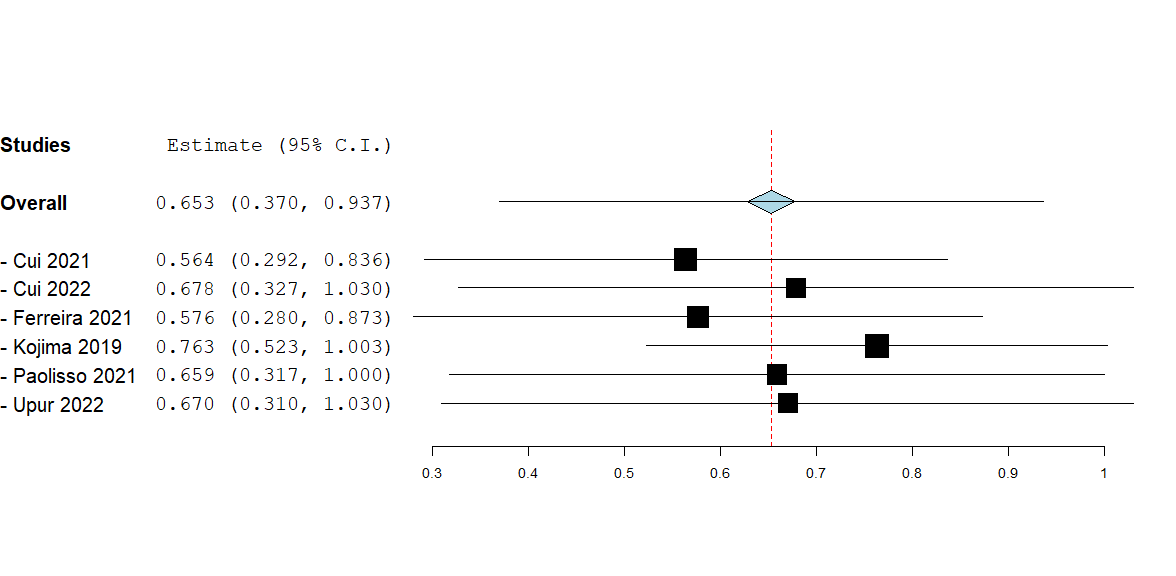


Supplementary figure 3: Leave-one-out analysis for mortality among diabetic patients using hazard ratio


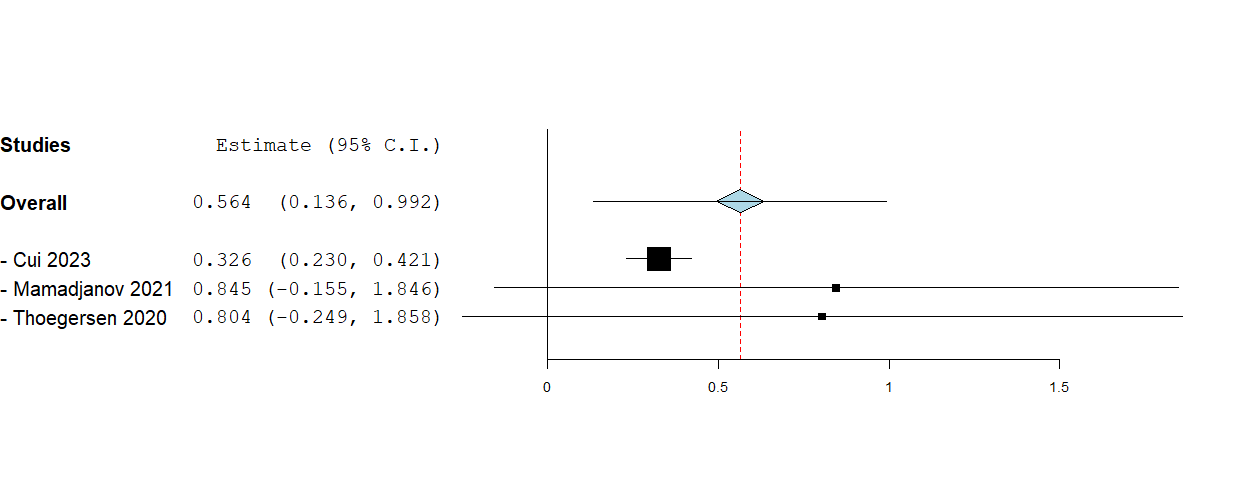


Supplementary figure 4: Leave-one-out analysis for mortality among diabetic patients using odds ratio


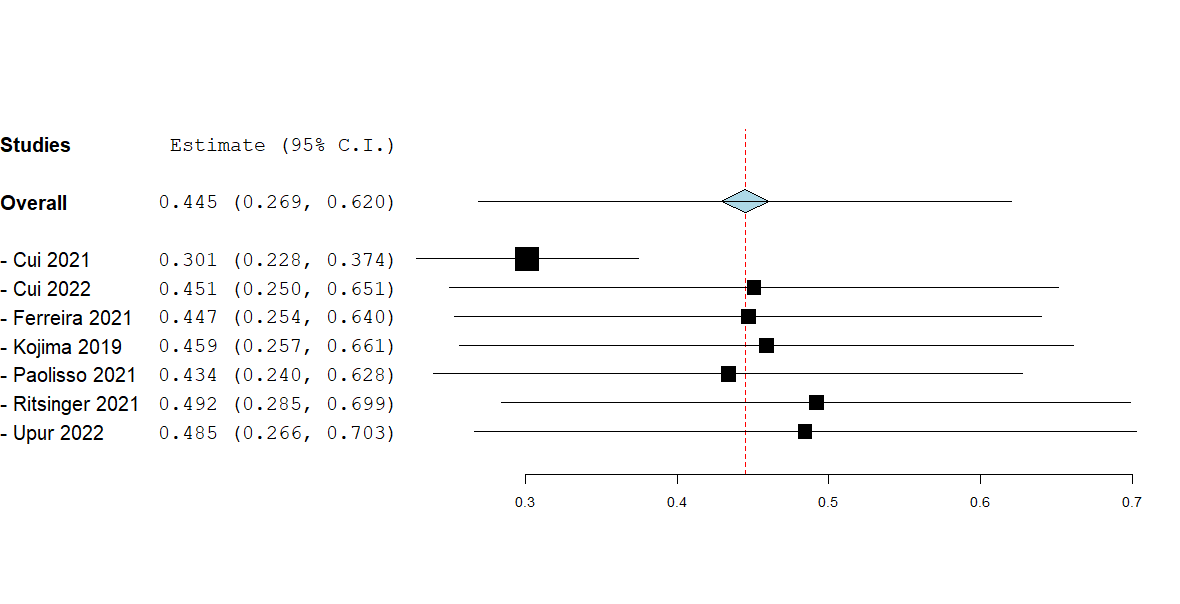


Supplementary figure 5: Leave-one-out analysis for mortality among non-diabetic patients using hazard ratio


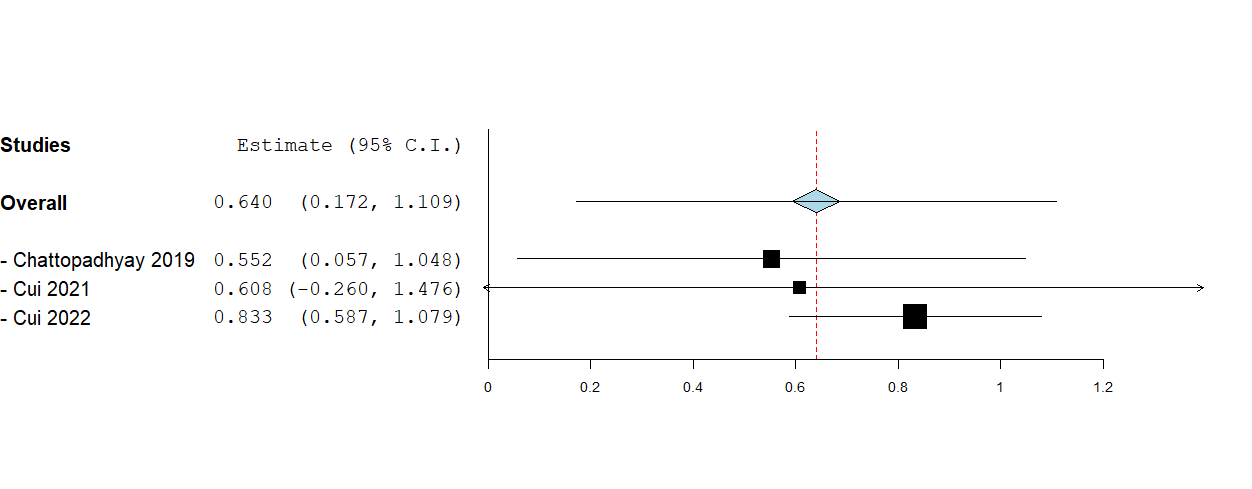


Supplementary figure 6: Leave-one-out analysis for MACE occurrence among diabetic patients using hazard ratio


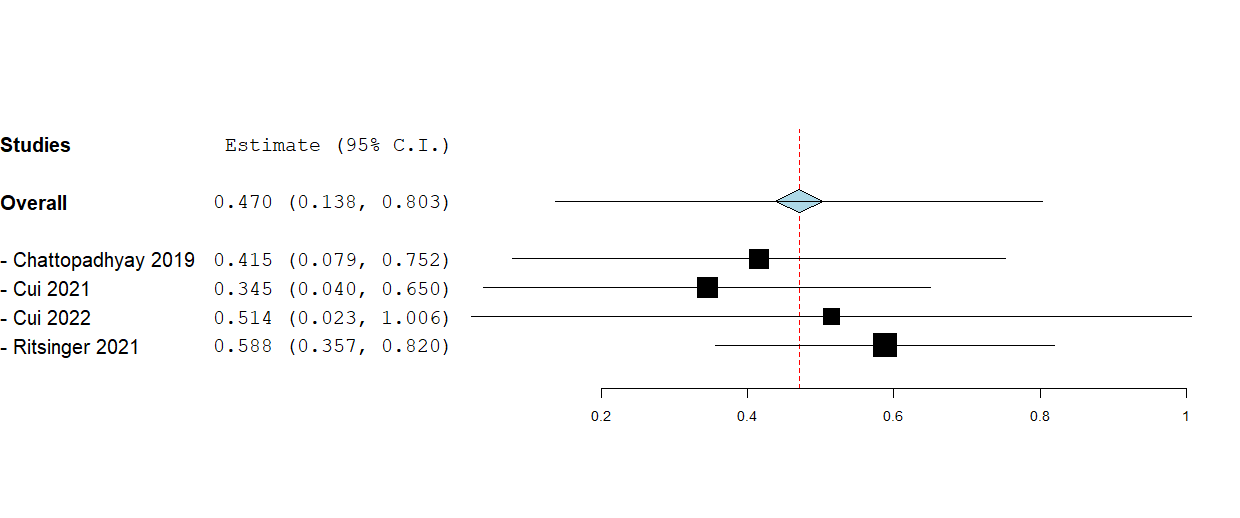


Supplementary figure 7: Leave-one-out analysis for MACE occurrence among non-diabetic patients using hazard ratio


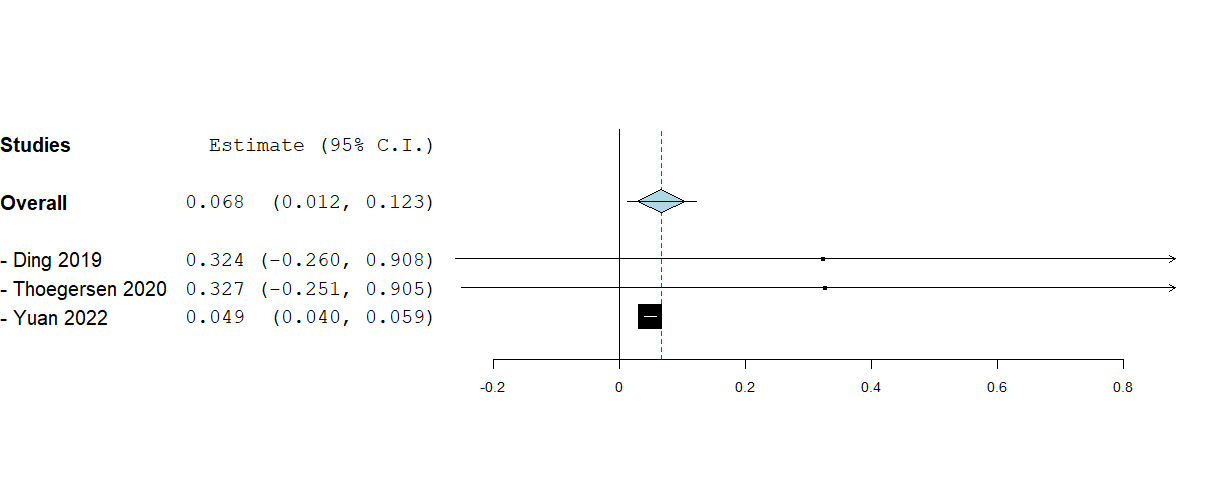


Supplementary figure 8: Leave-one-out analysis for association of age with mortality using odds ratio
