# Supplementary figures and images for: A systematic review and meta-analysis of the effect of hyperglycemia on admission for acute myocardial infarction in diabetic and non-diabetic patients
Source: Diabetol Metab Syndr. 2024 Sep 12;16:224. doi: 10.1186/s13098-024-01459-w (PMC11391676; doi:10.1186/s13098-024-01459-w)

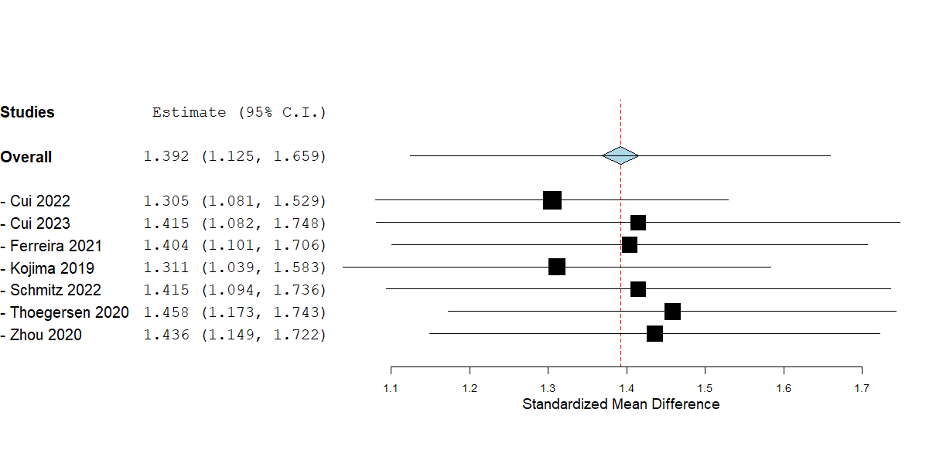

Supplement: Supplementary file 2 — Supplementary Material 2 [file 13098_2024_1459_MOESM2_ESM.png]

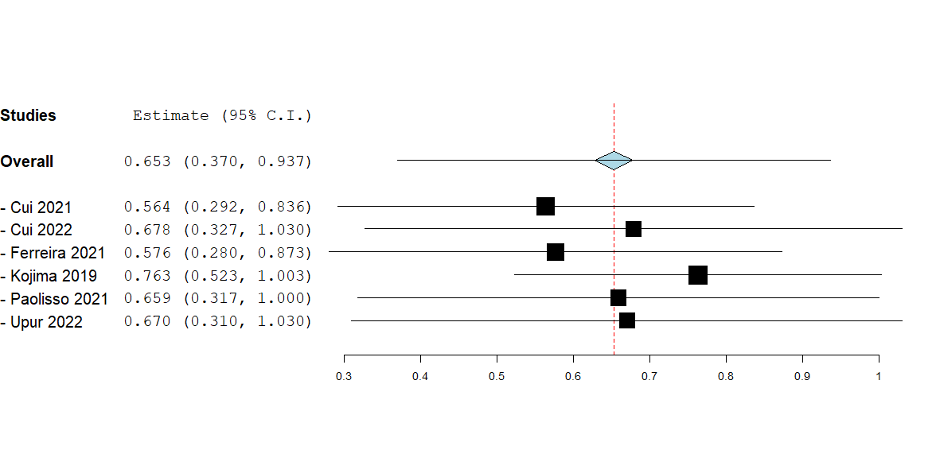

Supplement: Supplementary file 3 — Supplementary Material 3 [file 13098_2024_1459_MOESM3_ESM.png]

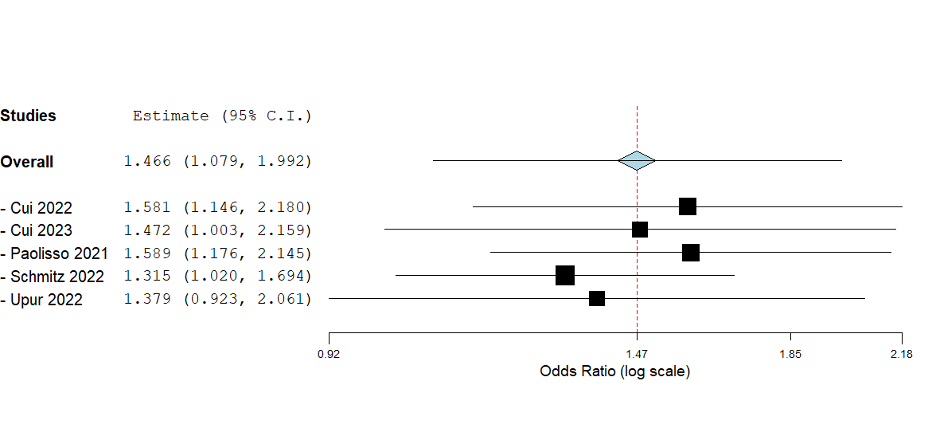

Supplement: Supplementary file 4 — Supplementary Material 4 [file 13098_2024_1459_MOESM4_ESM.png]

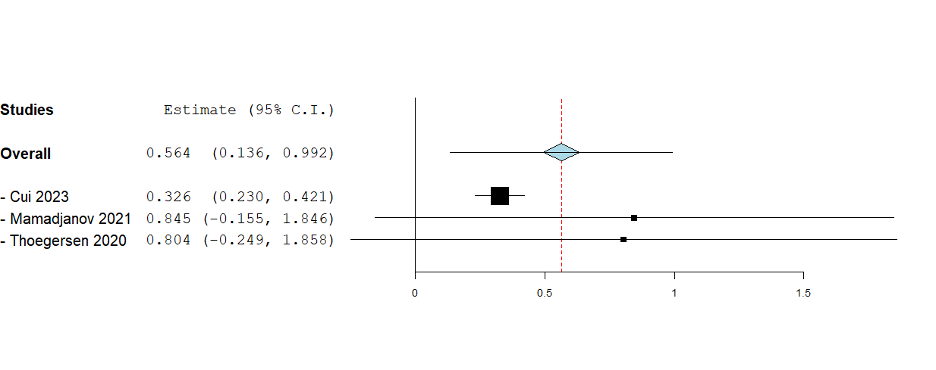

Supplement: Supplementary file 5 — Supplementary Material 5 [file 13098_2024_1459_MOESM5_ESM.png]

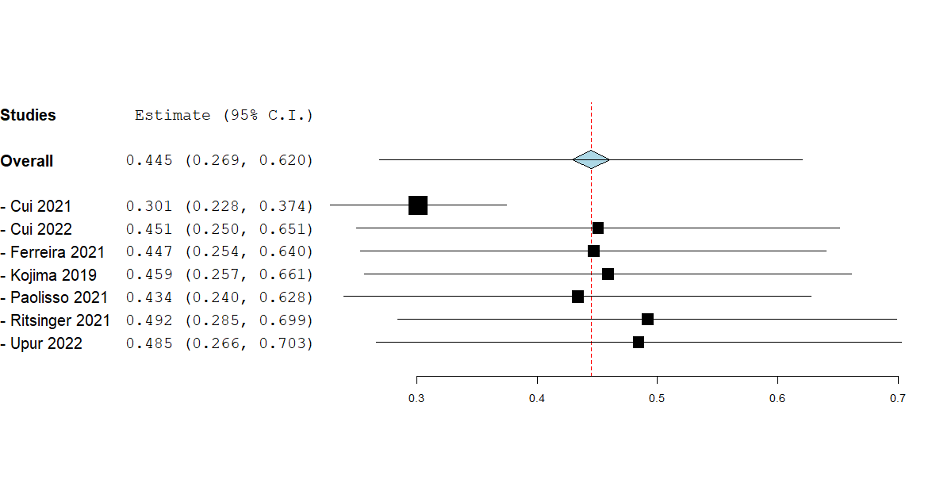

Supplement: Supplementary file 6 — Supplementary Material 6 [file 13098_2024_1459_MOESM6_ESM.png]

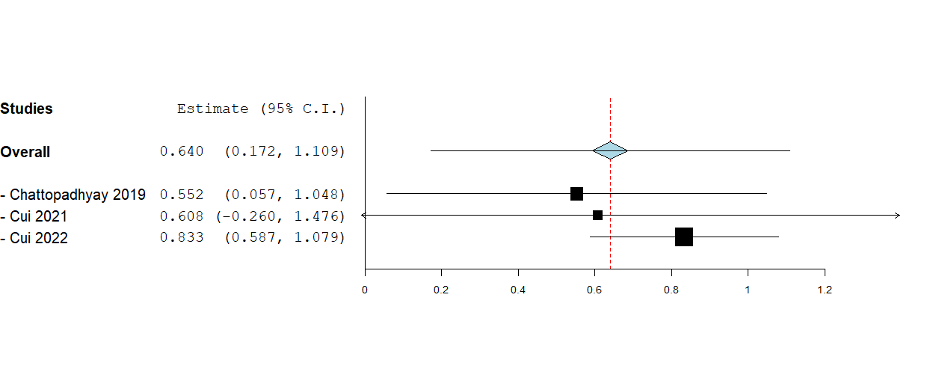

Supplement: Supplementary file 7 — Supplementary Material 7 [file 13098_2024_1459_MOESM7_ESM.png]

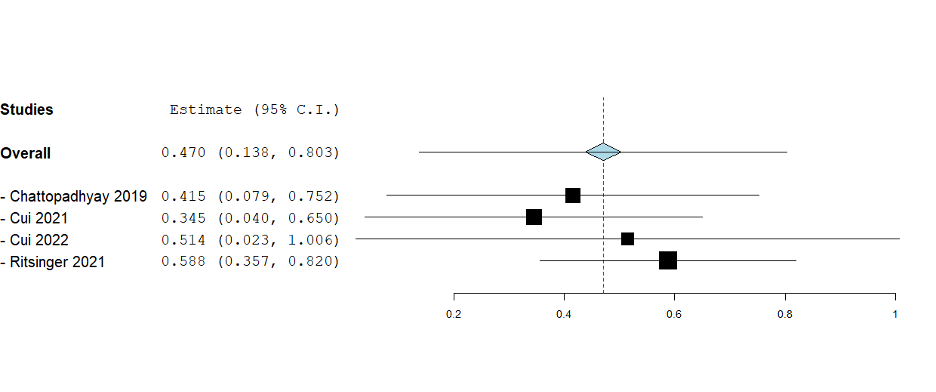

Supplement: Supplementary file 8 — Supplementary Material 8 [file 13098_2024_1459_MOESM8_ESM.png]

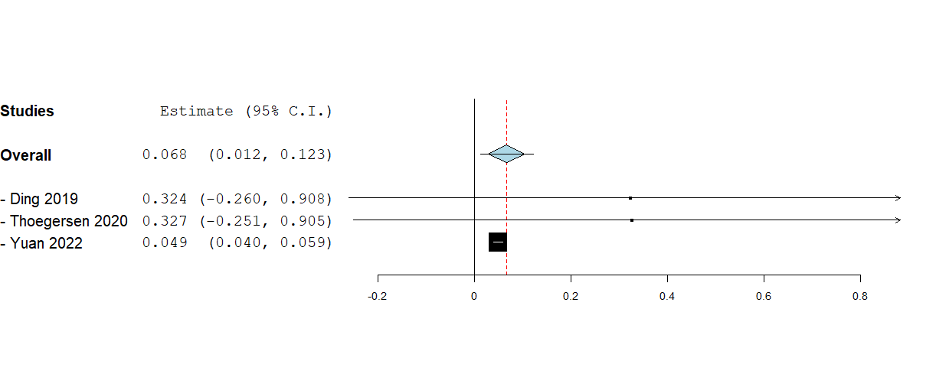

Supplement: Supplementary file 9 — Supplementary Material 9 [file 13098_2024_1459_MOESM9_ESM.png]
